# Multi-Junction Solar Cells and Nanoantennas

**DOI:** 10.3390/nano12183173

**Published:** 2022-09-13

**Authors:** João P. De Melo Cunha, Ricardo A. Marques Lameirinhas, João Paulo N. Torres

**Affiliations:** 1 Department of Electrical and Computer Engineering, Instituto Superior Técnico, 1049-001 Lisbon, Portugal; 2 Instituto de Telecomunicações, 1049-001 Lisbon, Portugal; 3 Academia Militar/CINAMIL, Av. Conde Castro Guimarães, 2720-113 Amadora, Portugal

**Keywords:** multi-junction solar cells, nanoantennas, optoelectronic devices, subwavelength structures, surface plasmon polaritons, photovoltaic technology, rectennas

## Abstract

Photovoltaic technology is currently at the heart of the energy transition in our pursuit to lean off fossil-fuel-based energy sources. Understanding the workings and trends of the technology is crucial, given the reality. With most conventional PV cells constrained by the Shockley–Queisser limit, new alternatives have been developed to surpass it. One of such variations are heterojunction cells, which, by combining different semiconductor materials, break free from the previous constraint, leveraging the advantages of both compounds. A subset of these cells are multi-junction cells, in their various configurations. These build upon the heterojunction concept, combining several junctions in a cell—a strategy that has placed them as the champions in terms of conversion efficiency. With the aim of modelling a multi-junction cell, several optic and optoelectronic models were developed using a Finite Element Tool. Following this, a study was conducted on the exciting and promising technology that are nanoantenna arrays, with the final goal of integrating both technologies. This research work aims to study the impact of the nanoantennas’ inclusion in an absorbing layer. It is concluded that, using nanoantennas, it is possible to concentrate electromagnetic radiation near their interfaces. The field’s profiles might be tuned using the nanoantennas’ geometrical parameters, which may lead to an increase in the obtained current.

## 1. Introduction

In a world where technology is being integrated in every part of our life, meeting the energetic demands of an ever-growing population creates a challenge. Traditional methods for energy production relied mainly on fossil fuels, be it coal, gas or even diesel. However, these energy sources create a strain on the environment due to releasing significant quantities of $CO_{2}$ into the atmosphere, which has caused an increase in the greenhouse effect, leading to the global warming experienced today. With the intent of maintaining global development while preserving the environmental balance, there has been a need to find alternative energy sources [1].

From our pursuit for cleaner and cheaper energy sources, the concept of renewable energy sources was created. These renewable sources of energy are infinite when put into perspective on a human timescale, meaning that they naturally replenish themselves. A good example of such a source is the Sun; this is where solar energy harvesting comes into play [1].

There are several solar energy harvesting methods, the most notable and widespread being based on photovoltaic (PV) technologies. There are a few reasons why PVs dominate the solar energy market. The core of the technology is based on one of the most abundant materials on Earth, Si, which has been intensively utilised and studied for integrated circuits manufacturing; therefore, technology for its production is cheap and the techniques are well known. Combining that with its stability, it becomes clear as to why the growth of the PV industry has been so significant in the last decades [1].

This rapid expansion led implicitly to an increase in competition in the market, and with the introduction of a scaled PV production, it in turn caused the levelised cost of energy (LCOE) of this technology to decrease significantly. As of today, PV technology presents the lowest LCOE, with projections of additional decreases in the years to come, estimating a cost of approximately USD 35.5$/MWh by 2021 [1,2].

There are still some challenges to tackle when it comes to photovoltaic (PV) technology, as most conventional PV solar cells—such as the homojunction crystalline Si—are limited in terms of their Power Conversion Efficiency (PCE) by the Shockley–Queisser limit, which sets the maximum at approximately 29.4% for Si-based cells [1,3,4]. As such, several new technologies have emerged, trying to surpass this value.

A possible way to overcome this limit is by utilising several junctions, as the aforementioned limit only applies to homojunction cells. Building on this approach, new types of cells were developed, the so-called heterojunction and multi-junction solar cells. With this new technology, new records of PCE were achieved, especially when applied in concentrator PV with a present record of 47.1% [1,5].

Another way to overcome the limit—this one, so far, only in theory—is with a completely different type of solar harvesting technology. Thus, the concept of a rectenna, or rectifying antenna, was introduced. The overall concept of a rectenna is rather simple, it is an antenna in series with a rectifier. However, to rectify a certain wavelength, one has to size the antenna accordingly as well as the operating frequency of the rectifier.

If we consider the Sun’s spectral irradiance, most of its energy is in the visible and near-infrared region of the spectrum. So, for a rectenna to harvest solar energy it would need to interact with wavelengths approximately of 380 nm to 700 nm, meaning it would need to be in the nanoscale and its rectifier has to operate at a frequency in the order of THz.

Due to improvements on fabrication techniques, building structures in the nanoscale no longer imposes an impossibility to this technology; however, the frequency of operation needed for the rectifier does. As of this moment, there is still no rectifier that can efficiently operate in the THz range; therefore, rectennas for solar energy harvest remain a concept. This does not imply that the underlying technology has no practical application. Nanoantennas have been proposed for numerous applications, from molecular detection to imaging and light absorption. In an attempt to explore more options, the combination of nanoantennas and heterojunction solar cells will be explored [1].

This research work aims to study the impact of the nanoantennas’ inclusion in an absorbing layer. By tuning the nanoantennas’ geometrical parameters, it should be possible to obtain different radiation patterns, which may lead to an increase in the obtained current.

## 2. Nanoantennas

With the development of fabrication techniques, the size of the devices that were possible to reliably reproduce decreased, this enabled the creation of new branches of scientific investigation that aimed to study possible applications for devices of reduced dimensions. Nano-optics is an example of such a branch, which aims to study the interaction of light–matter with nanoscale devices, such as nanoantennas [6,7,8].

Nanoantennas can have many uses such as imaging, spectroscopy and the development of sensors. However, while the subject of the next section implies there is another possible application for nanoantennas, that so far has proven elusive due to some technological constraints still present. Nanoantennas can, theoretically, serve as an alternative energy source when applied to solar energy harvesting. This device is, at its core, a rectifying antenna designed to work at frequencies present in the solar spectrum, giving it the name solar rectenna [6,7].

The concept of an antenna for solar energy harvesting was introduced after successful experimentation in the 1960s with rectifying antennas to power devices in a wireless configuration using a microwave source [9]. The idea was to apply the proven concept to higher frequencies, present in the visible and infrared regions of the solar spectrum, since the majority of the Sun’s radiated energy is located there.

To work in that region, the antenna would need to be scaled down to the nanoscale, since the size of the antenna is linked with the targeted wavelength. For the visible and infrared regions of the spectra the frequency of the electromagnetic waves is in the order of THz, meaning wavelengths of μm to nm, from far in the infrared region to near the visible. The other aspect to take into account is the corresponding rectifying element, since the antenna must be coupled with a rectifier in order to convert from alternating current to direct, allowing for a more manageable operation. As the incident electromagnetic wave determines the frequency of the oscillating free charges [7,10], the frequency of the rectifying element must match the frequency of the incident wave. Therefore, the rectifying frequency must be in the THz range as well.

When the concept of the solar rectenna was first theorised, light–matter interaction at the nanoscale was not clear, as fabrication techniques in the nanoscale were yet to be mastered [9]. So, scaling down common designs of antennas used in the communications field to the optical range was required, as was intended initially, so as to take into account not only the wavelength but also the unique material interactions that occur at such frequencies, as they affect the behaviour of the antenna.

As the frequency increases, the nature of metals changes, nearing that of a semiconductor since at optical frequencies the perfect conductor approximation is no longer valid, due to the penetration of the electromagnetic waves in the material and the increased dissipation [6,7,9,10]. This penetration of the material by the electromagnetic wave results in oscillation of the conduction electrons, giving rise to formation of plasmons and inducing resonant effects that were not present at lower frequencies.

A plasmon is defined as a quasiparticle—a quantisation of a plasma oscillation—that arises from the excitation of the free electron by the penetration of the incident electromagnetic waves. When the oscillation of the plasmon matches that of the incident photons, another quasiparticle is created, a polariton [11]. When the plasmons are confined to the interface of a metal and a semiconductor, they are defined as surface plasmons, of which there are two types, surface plasmon polaritons and localised surface plasmons. The first, as mentioned before, is the coupling of a photon and a plasmon in the surface of the metal, allowing for propagation at the interface. The second is when the plasmon interacts with nanoparticles; in this case, the electromagnetic wave interacts with the electrons in the electron cloud of the particles, originating a localised oscillation, displacing it according to the variations in the electric field. The resonance effects that arise due to the nanoparticles lead to field amplification in the particle and near its position [12].

Spatially confining the field to harness the advantage of this phenomena is then the focal point of nanoantenna design. Further, as mentioned previously, a branch of nanoantenna designs are scaled-down versions of antennas used in the communication field, such as the Yagi–Uda, but there are several more.

The wide variety of metallic nanoantennas stem from studies performed on metallic nanoparticles in the 1920s by Edward Hutchinson Synge and later with Fisher and Pohl in the 1980s that first theorised and then showed that these metallic nanoparticles could function as an antenna [9,13]. The characteristics of such a device depends on several aspects, such as its geometry, material composition, and the surrounding dielectric; however, it marks one of the simplest nanoantenna designs achievable, known as the plasmonic monopole [6,8,13]. Not long after the discovery of the plasmonic monopoles, it was verified that the field amplification was more significant if a dipole was used, since the gap between two plasmonic nanoparticles allowed for a greater spatial field confinement. There are several noteworthy designs in the dipole category, for example, the plasmonic bow tie, as its shape mirrors that of its garment counterpart.

## 3. Model and Simulations

### 3.1. Multi-Junction Cell and Characteristics

With efficiency as the primary criteria, the cell chosen to model in the present work is the one described in [5], the record holder in terms of PCE. Having chosen the cell, a layer-based model was developed on a Finite Element Tool, with recourse to MATLAB to perform auxiliary computations and to aid in data visualisation.

**Figure 1:**
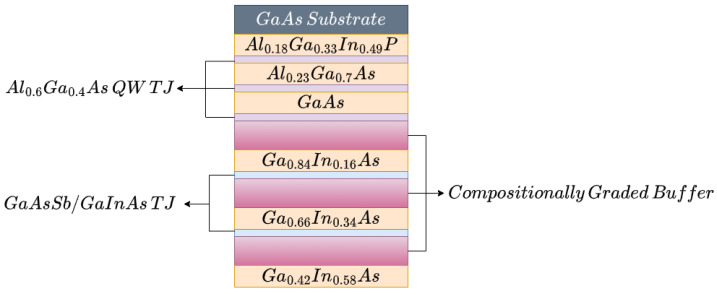
Depiction of the multi-junction cell.

In terms of its characteristics, the cell described in [5] is a six-layer inverse metamorphic device, as depicted in Figure 1. It has the first two junctions’ lattices matched to GaAs, and the rest are increasingly mismatched [5]. In a metamorphic optical device such as this, the addition of a CGB between the mismatched layers is required to smooth the transition from one layer to the next. Further, present in the junctions are the so called Tunnel Junction (TJ), tasked with passing current between adjacent junctions with minimal losses.

Regarding the developed model, a simplified approach had to be taken, with a particular focus on the absorber layers. In essence, by disregarding the influence of the CGB and TJ present in the original optical device, it was assumed that they performed ideally. This choice had to be taken, as a fully detailed model would require greater computational resources than those available at the time. Nonetheless, the simplified model still allows for an in-depth study of the optical properties, with an emphasis on the optical characteristics of each compound and the contribution of each layer for the formation of current, as will be shown further ahead.

### 3.2. Cell Optical Model

Following the proposed methodology, the first step is the construction of the layer model for optical study. For each absorber layer, a 2D representation was designed, with the first analysis focused on obtaining its absorption coefficient α(ω).

As mentioned previously, the optical properties of the materials vary, depending on the frequency of the incident radiation. This relation is conveyed in ϵ(ω), the complex dielectric function [14], as in Expression (1), with the refractive index, extinction coefficient and absorption coefficient all linked to it.

(1) $$\epsilon \left(\omega \right)=\epsilon_{1}\left(\omega \right)+i\epsilon_{2}\left(\omega \right)$$

In the case of the refractive index, the connection is given by Expression (2)

(2) $$\bar{n}\left(\omega \right)=n\left(\omega \right)+ik\left(\omega \right)=\epsilon {\left(\omega \right)}^{\frac{1}{2}}$$

where $\bar{n}$ is the complex refractive index, from which the ideal refractive index *n* and extinction coefficient *k* can be extracted.

Given the objectives of the present work, and in particular, at this stage, the extinction coefficient plays an important role since it determines the attenuation of a medium.

From Beer’s law, describing the intensity attenuation of a beam passing through a medium, the link with the extinction coefficient can be expressed [15], as in Expression (3).

(3) $$\alpha \left(\omega \right)=\frac{\omega k(\omega )}{c}=\frac{4\pi k(\omega )}{\lambda }$$

With the absorption coefficient α(ω), for the same path length, the differences displayed by each compounds gives an indication of their influence on the electromagnetic field, and therefore, their role in the multi-junction device.

In order to build a 2D layer model, data regarding the dielectric function and refractive index of the different compounds were needed.

There are several approaches in terms of modelling experimental data. The ones used in this work follow the semiempirical route.

For the first absorber layer, $Al_{0.18}Ga_{0.33}In_{0.49}P$, Adachi’s dielectric function model was used [16]; for the remaining layers, the model described in the works of Cuevas et al. [17] was used. Both models adopt a hybrid semiempirical approach, combining the advantages that a standard critical point model provides for lower energy levels, and a damped oscillator model for higher energy levels. Greater detail regarding the advantages and disadvantages of several model types can be found in literature [14,16,17,18,19,20,21].

Accordingly to [17], it is possible to determine the critical points taken into account by the semiempirical model, exemplifying in this case the energy band structure of $Al_{x}Ga_{1-x}Sb$. The most relevant contributions are, $E_{0}$ for direct gap transitions, $E_{0}+\Delta_{0}$ for the spin–orbit split component, $E_{1}$ and $E_{1}+\Delta_{1}$ spin–orbit split doublet, $E_{2}$ for the higher lying peaks and $E_{g}^{ID}$ for indirect gap transitions. Combining all of these contributions gives the spectral dependence of the dielectric response.

Following [17], the dielectric response would be obtained with the following expressions.

The contributions of $E_{0}$ and $E_{0}+\Delta_{0}$ transitions is obtained with Expression (4)

(4) $$\epsilon_{1}\left(v\right)=AE_{0}^{-1.5}\left(g\left(\frac{hv+i\Gamma }{E_{0}}\right)+0.5\left(\frac{E_{0}}{E_{0}+\Delta_{0}}\right)g\left(\frac{hv+i\Gamma }{E_{0}+\Delta_{0}}\right)\right)$$

where *A* is a strength parameter, Γ is a dampening constant and *g*(*x*) is

(5) $$g\left(x\right)=x^{-2}\left({\left(1+x\right)}^{\frac{1}{2}}-{\left(1-x\right)}^{\frac{1}{2}}\right)$$

For $E_{1}$ and $E_{1}+\Delta_{1}$, the dielectric contribution follows (6)

(6) $$\epsilon_{2}\left(v\right)=-B_{1}{\left(\frac{hv+i\Gamma }{E_{1}}\right)}^{-2}ln\left(1-{\left(\frac{hv+i\Gamma }{E_{1}}\right)}^{2}\right)-B_{2}{\left(\frac{hv+i\Gamma }{E_{1}+\Delta_{1}}\right)}^{-2}ln\left(1-{\left(\frac{hv+i\Gamma }{E_{1}+\Delta_{1}}\right)}^{2}\right)$$

with $B_{1}$ and $B_{2}$ as strength parameters, determined by Expressions (7) and (8), respectively

(7) $$B_{1}=44\frac{E_{1}+\frac{\Delta_{1}}{3}}{a_{lc}E_{1}^{2}}$$

(8) $$B_{2}=44\frac{E_{1}+\frac{2\Delta_{1}}{3}}{a_{lc}{\left(E_{1}^{2}+\Delta_{1}\right)}^{2}}$$

with $a_{lc}$ as the lattice constant.

The third contribution to the dielectric response is that of $E_{2}$, which corresponds to a well-defined critical point, and as such, is characterised by a damped harmonic oscillator, as expressed in (9)

(9) $$\epsilon_{3}\left(v\right)=\frac{CE_{2}^{2}}{E_{2}^{2}-{\left(hv\right)}^{2}-ihv\Gamma }$$

with *C* as a nondimensional strength parameter.

Lastly, the contributions resulting from indirect band gap transitions, $E_{g}^{ID}$, are as in Expression (10)

(10) $$\begin{array}{c} \epsilon_{4}\left(v\right)=-\frac{2D}{\pi }{\left(\frac{E_{g}^{ID}}{hv+i\Gamma }\right)}^{2}ln\left(\frac{E_{c}}{E_{g}^{ID}}\right)+\frac{D}{\pi }{\left(1+\left(\frac{E_{g}^{ID}}{hv+i\Gamma }\right)\right)}^{2}ln\left(\frac{hv+i\Gamma +E_{c}}{hv+i\Gamma +E_{g}^{ID}}\right)\\ +\frac{D}{\pi }{\left(1-\left(\frac{E_{g}^{ID}}{hv+i\Gamma }\right)\right)}^{2}ln\left(\frac{hv+i\Gamma -E_{c}}{hv+i\Gamma -E_{g}^{ID}}\right)) \end{array}$$

once more with *D* as a strength parameter, in this case, independent of photon energy; additionally, $E_{c}$, as a high energy cutoff, is assumed to be equal to $E_{1}$.

The complex dielectric response is obtained by simply adding the previous four contributions, as in Expressions (4), (6), (9) and (10), resulting in (11). First, there are some auxiliary computations that need to be performed, and given that some parameters show a dependence on temperature, several steps are needed.

(11) $$\epsilon =\epsilon_{1}+\epsilon_{2}+\epsilon_{3}+\epsilon_{4}$$

The dependence on temperature is due to lattice expansion and cation–phonon interactions [17]. To account for these changes, a correction to the critical points is performed, with recourse to the Varhsni Equation (12).

(12) $$E\left(T\right)=E\left(T=0\right)-\frac{\delta T^{2}}{T+\beta }$$

where E(T = 0) is an extrapolated energy value at absolute zero, with δ and β as fitting constants. The used values were from [17]. It should also be noted that the temperature value chosen for all the calculations performed was T = 300 K, an average room temperature value.

Another phenomenon that requires some parameter adjustment is sharpening of the higher energy peaks at lower temperatures, namely, $E_{1}$, $E_{1}+\Delta_{1}$ and $E_{2}$ peaks. This sharpening is correlated with a lifetime broadening reduction that, in turn, can be expressed as in (13)

(13) $$\Gamma \left(T\right)=\Gamma_{L}+\gamma T$$

where $\Gamma_{L}$ and γ are fitted constants. Once more, the used values are from [17].

The lattice expansion remains to be acknowledged. As is evident in Expression (14), the temperature chosen to perform these computations is tied to the lattice constant, as it negates the second term of the expression, leaving its reference value unaltered. For the lattice expansion, its relation with temperature is conveyed in (14).

(14) $$a_{lc}\left(T\right)=a_{lc}\left(300\right)+k\left(T-300\right)$$

Although most parameters suffered with temperature, some had changes that so were negligible that they could be assumed temperature insensitive.

With the temperature dependence of the parameters accounted for, and as the goal of [17] was to determine a composition dependent model for quaternary III–V semiconductors, each parameter now suffers additional corrections, to account for these changes.

It becomes clear that the values shown are in regard to binary alloys. To obtain the parameters for a ternary or quaternary alloy, as is needed in this case, an interpolation method is required.

To obtain the parameters for a ternary or quaternary alloy, as is needed in this case, an interpolation method is required. The method followed in this work was in Expression (15), Vegard’s Law, for a $A_{x}B_{1-x}C$ type ternary, with $B_{ac}$ and $B_{bc}$ as the binary alloy parameter values and C a bowing constant, to account for lattice deviations.

(15) $$T_{ABC}\left(x\right)=xB_{ac}+\left(1-x\right)B_{bc}-x\left(1-x\right)C$$

The bowing constant values used to correct the parameters of the binary alloys are obtained also in [17].

Given that all the dependencies of the model have been described, the flow of the computations performed should also be clarified. In this sense, with the ternary alloy $Al_{0.23}Ga_{0.77}As$ as an example, to compute the correct value for the dielectric response for a given frequency, the computations are as follows.

Firstly, it is necessary to account for the temperature dependence of the peaks by correcting the energy values of the critical points with Expression (12) for peak sharpening, with Expression (13) and, lastly, with (14) for lattice expansion.

These three steps correct the binary parameter values of AlGa and GaAs, in this case, for all the variations due to temperature.

The next stage of computations is the composition dependence, to obtain the correct values for the ternary alloy. This is accomplished following Vegard’s law (15), which for $Al_{0.23}Ga_{0.77}As$ requires *x* = 0.77 and the corresponding bowing constants.

Following Vegard’s Law, all the critical point energies are not only corrected for temperature but also for composition. Therefore, determining the dielectric response is next, from Expression (4) to (11).

As the developed model required the optical constant values, these are obtained following Expressions (16) and (17) for the real and imaginary parts of the complex refractive index, respectively.

(16) $$n={\left(\frac{{\left(\epsilon_{1}^{2}+\epsilon_{2}^{2}\right)}^{\frac{1}{2}}+\epsilon_{1}}{2}\right)}^{\frac{1}{2}}$$

(17) $$k={\left(\frac{{\left(\epsilon_{1}^{2}+\epsilon_{2}^{2}\right)}^{\frac{1}{2}}-\epsilon_{1}}{2}\right)}^{\frac{1}{2}}$$

The following subsections display the results for all the absorption layers in terms of the optical constants and some snapshots of the electric field intensity in the different layers.

It should be noted that the required parameter values needed to compute all the previous expressions can be found in [17].

**Figure 2:**
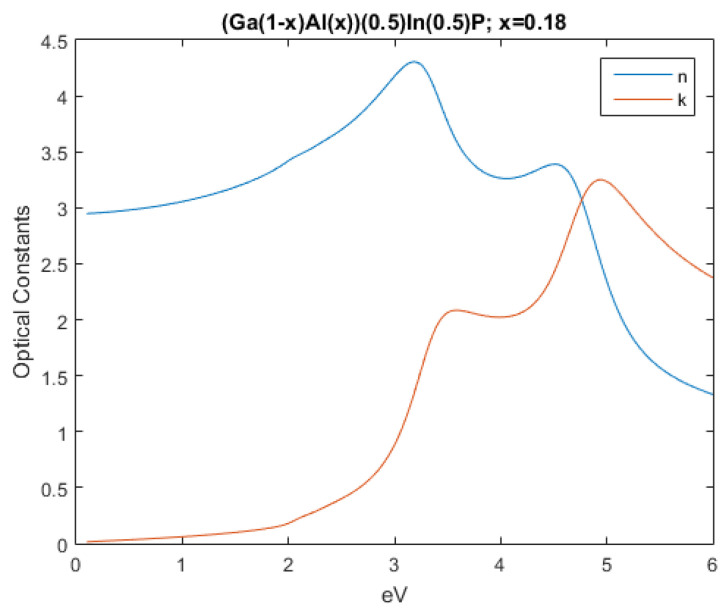
Real and imaginary optical constants of $Al_{0.18}Ga_{0.33}In_{0.49}P$.

Following an identical procedure as the one described earlier, the optical constants of $Al_{0.18}Ga_{0.33}In_{0.49}P$ were obtained, resulting in the variation displayed in Figure 2.

With these values, an optical study was performed where the influence of the layer in the electric field was the focal point.

**Figure 3:**
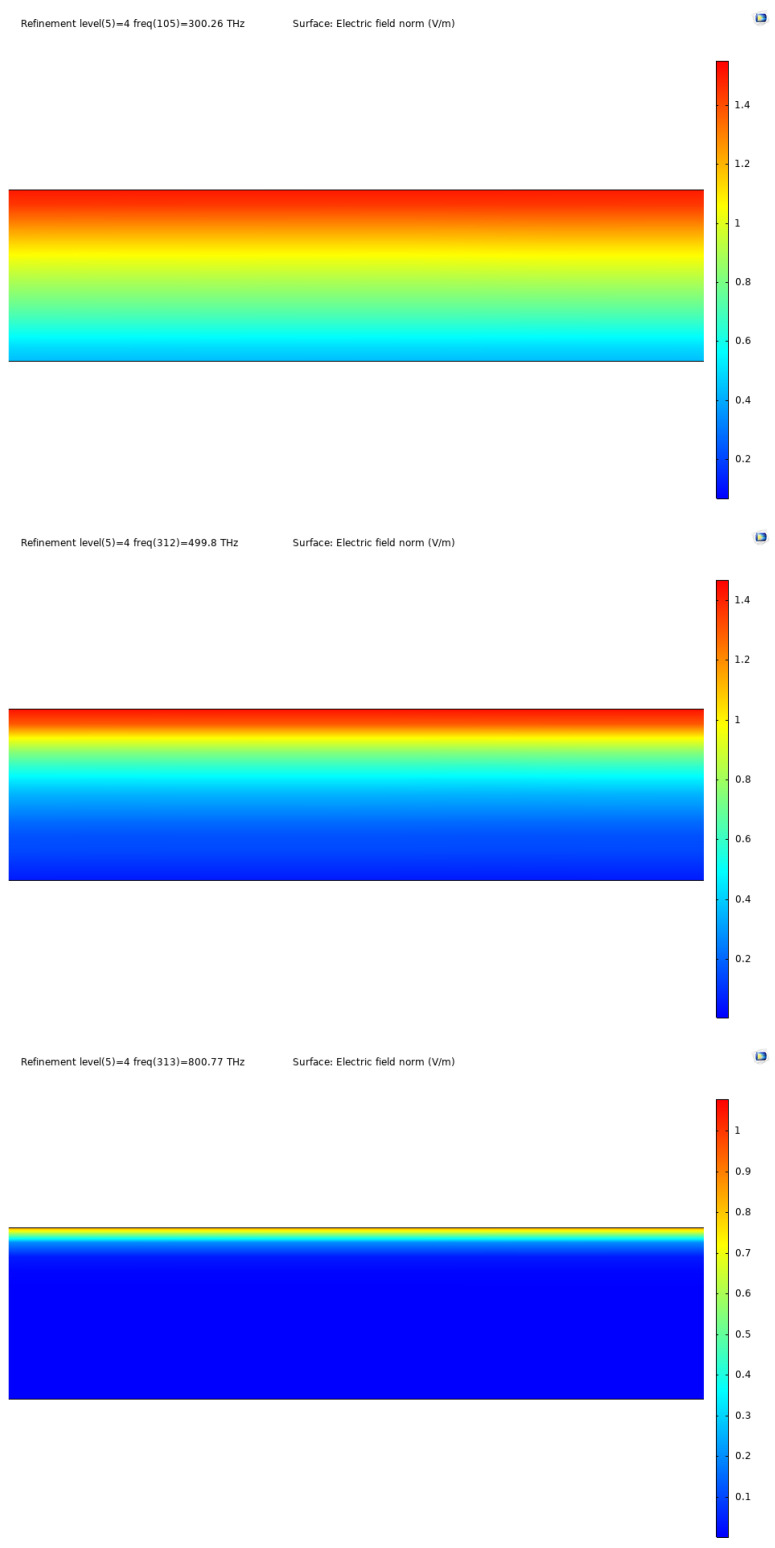
Electric field norm for different frequencies for $Al_{0.18}Ga_{0.33}In_{0.49}P$, at 300 THz, 500 THz and 800 THz.

As is clear in Figure 3, the attenuation of the layer differs in accordance with frequency, as expected, and the attenuation increases with an increase in frequency, a fundamental characteristic for this type of device. For reference, further into the higher energy spectrum, the attenuation is as displayed in Figure 3.

**Figure 4:**
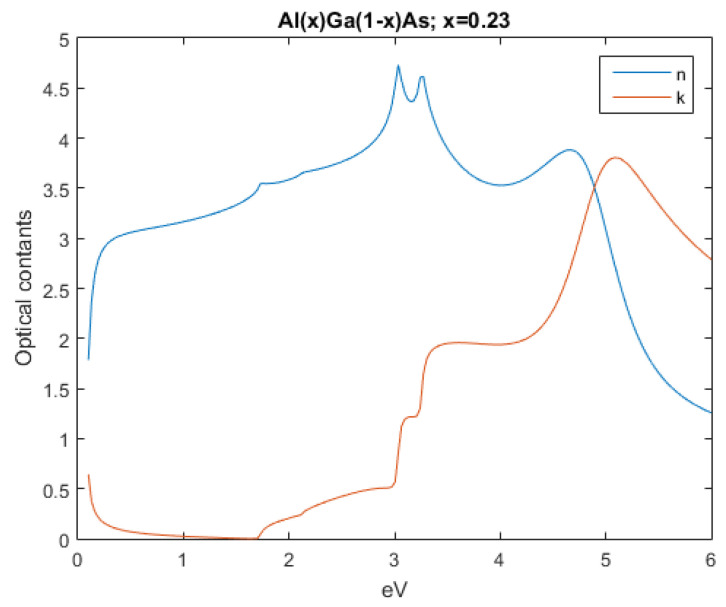
Real and imaginary optical constants of $Al_{0.23}Ga_{0.77}As$.

**Figure 5:**
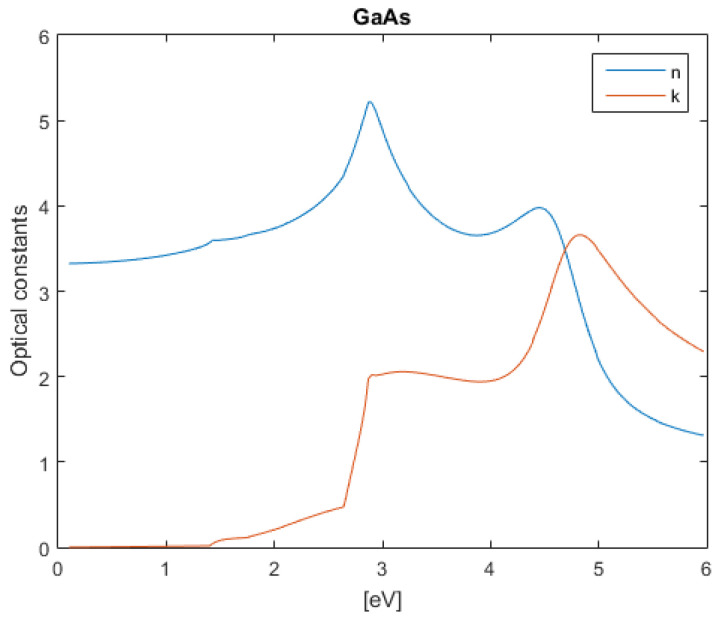
Real and imaginary optical constants of GaAs.

**Figure 6:**
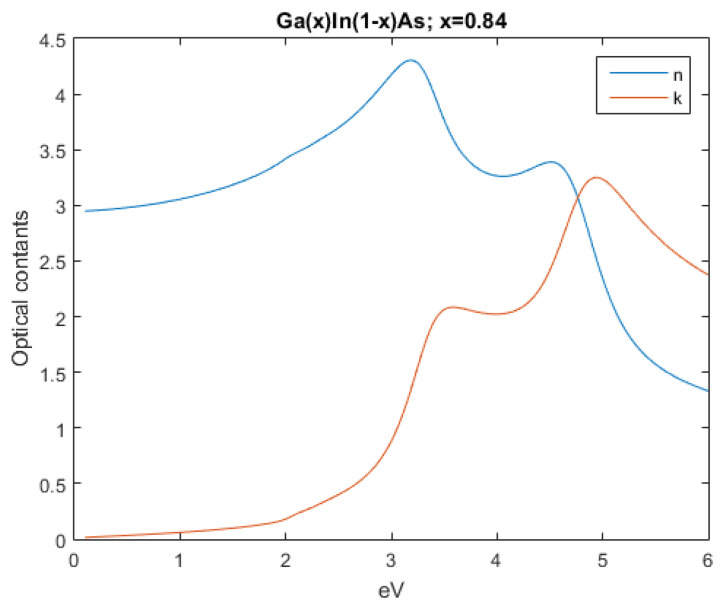
Real and imaginary optical constants of $Ga_{0.84}In_{0.16}As$.

**Figure 7:**
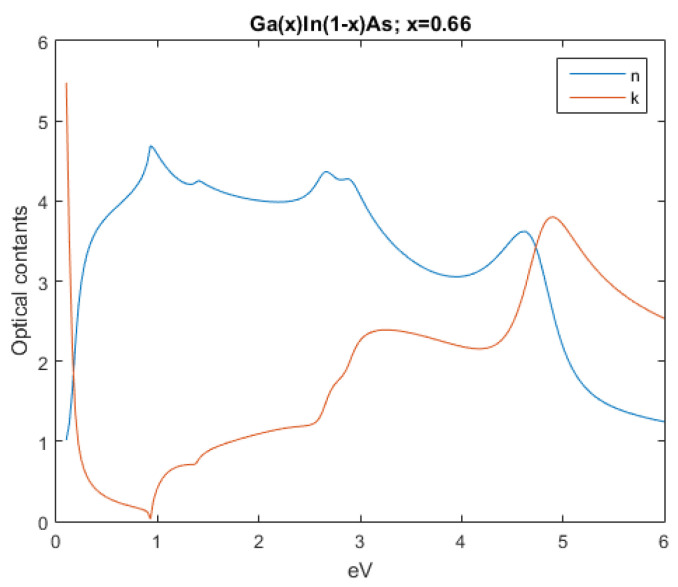
Real and imaginary optical constants of $Ga_{0.66}In_{0.34}As$.

**Figure 8:**
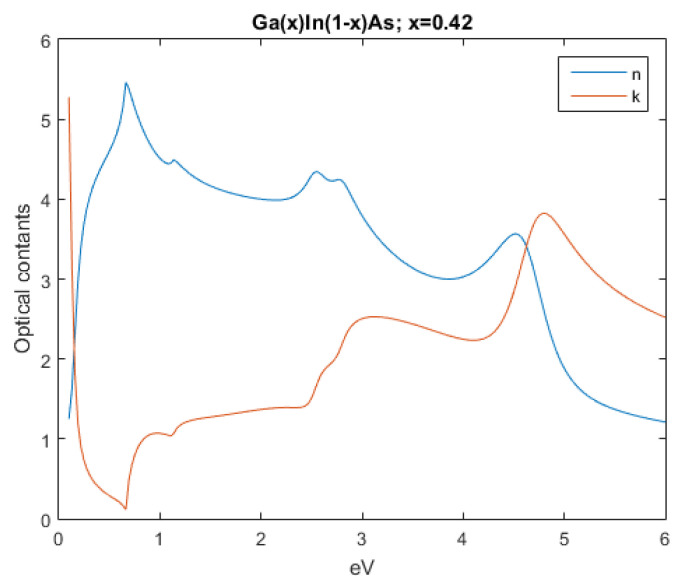
Real and imaginary optical constants of $Ga_{0.42}In_{0.58}As$.

The subsequent figures, from Figure 4, Figure 5, Figure 6, Figure 7 and Figure 8, show the complex refractive index components of the other semiconductors. The respective radiation/field patterns are presented in Appendix A.

### 3.3. Attenuation

The optical device in study is a multi-junction PV cell. To obtain the binned absorption of the spectrum, a good absorption layer, from an optical standpoint, should interact with the incident radiation until a certain frequency threshold, beyond which it should allow the radiation to pass through the layer undisturbed. This should hold especially true the higher the layer is in regard to cell, with the exception of the last layer, which is tasked with absorbing the remnant spectra.

Following this basis, some trend in the field intensity across the layers should become apparent.

**Figure A4:**
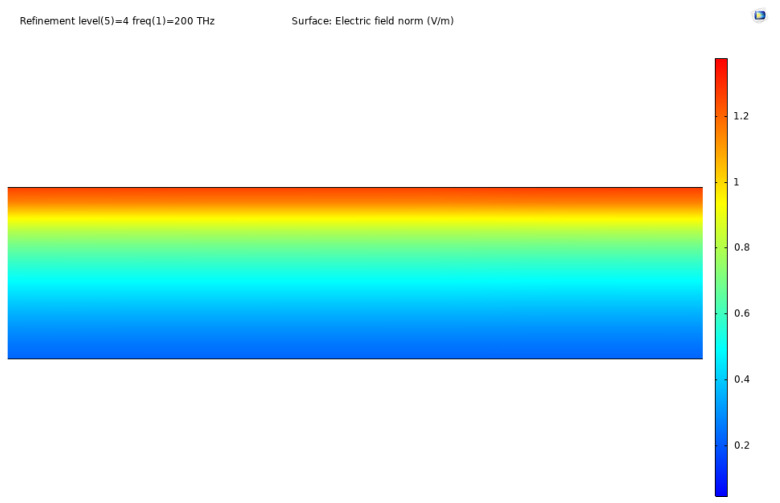

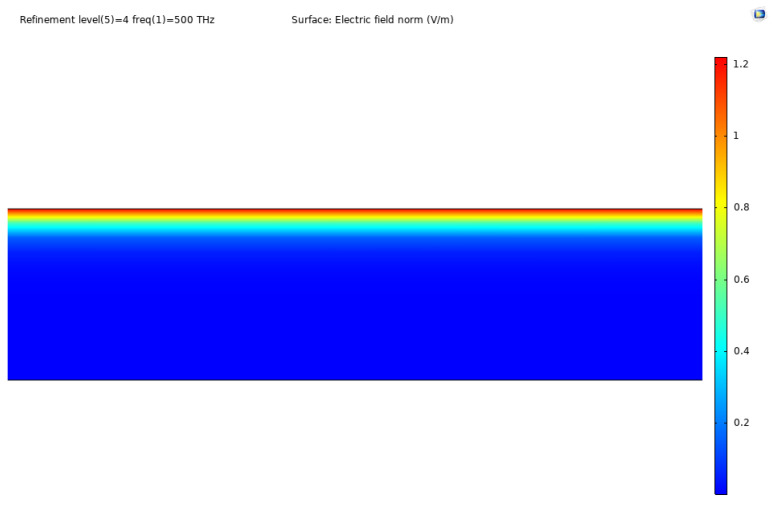
Electric field norm of $Ga_{0.66}In_{0.34}As$ at 200 THz and 500 THz.

**Figure A5:**
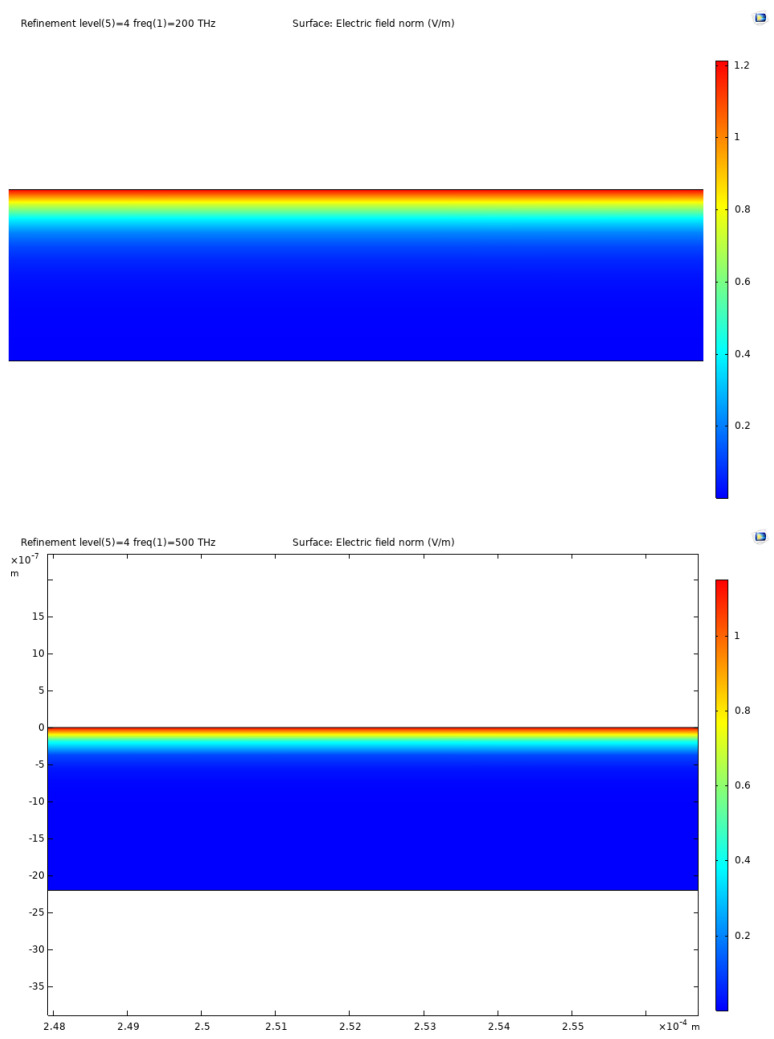
Electric field norm of $Ga_{0.66}In_{0.34}As$ at 200 THz and 500 THz.

Comparing, for example, Figure A4 and Figure A5, the differences are clear. The top layer, $Ga_{0.66}In_{0.34}As$, allows for lower frequencies to transverse it while the bottom layer, $Ga_{0.42}In_{0.58}As$, where the attenuation coefficient is higher, absorbs it.

In regard to the attenuation coefficient, its relation with the electric field from a theoretical standpoint is demonstrated following Beer’s Law, (18).

(18) $$I=I_{0}e^{-\alpha x}$$

with *I* as beam intensity and x the path length in the medium. For a plane wave, the beam intensity is related to the electric field intensity from *S*, as in (19).

(19) $$S=E\;\cdot \;H=\frac{{\left|E\right|}^{2}}{\eta }$$

Factoring (18) in relation to α, and considering (19), results in (20), which correlates the field intensity with the path length and the attenuation coefficient.

(20) $$\alpha =-\frac{2}{x}ln\left(\frac{\left|E\right|}{|E_{0}|}\right)$$

**Figure 9:**
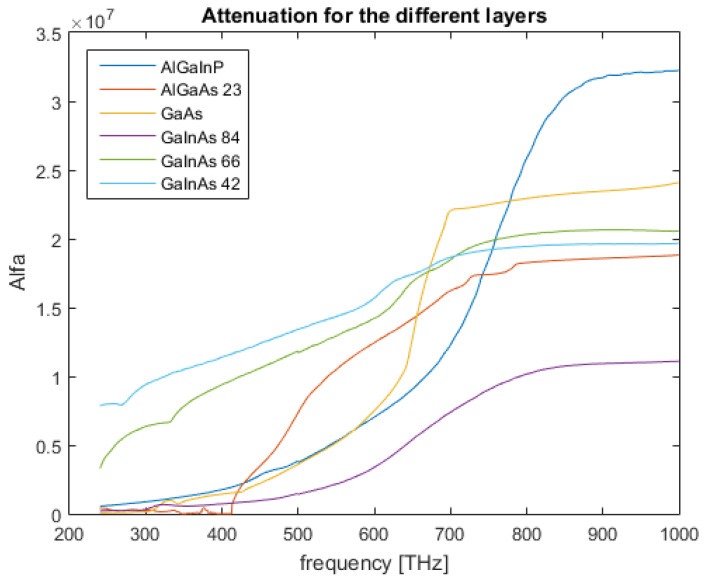
Attenuation of the different layers.

Experimentally, the attenuation coefficient was obtained by probing the field intensity at the surface of the layer and at the bottom, upon exit, while maintaining a fixed path length for all the layers and frequencies. The data were then exported to MATLAB so that (20) could be factored in. Performing a frequency sweep on each layer and computing its attenuation across the interval results in Figure 9.

A careful analysis of the graphs displayed in Figure 9 corroborates the attenuation profile described earlier, with an emphasis on higher frequency attenuation in the top layers coupled with a decrease for lower ones, and higher attenuation on said spectra for the lower-laying layers.

### 3.4. Cell Optoelectronic Model

With the optical profile of the cell complete, the next step is the optoelectronic model. Given the device, current formation and the correlation with the incident electromagnetic field warrants particular attention, and will therefore be the focal point of the present section.

The developed model for this analysis can be viewed as a complement to the previous one, with added information regarding carrier mobility, density of states and electron affinity.

Following the data provided in [22], it was possible to describe the responses of $Al_{0.23}Ga_{0.77}As$ and $Ga_{0.42}In_{0.58}As$. For $Ga_{0.66}In_{0.34}As$ and $Ga_{0.84}In_{0.16}As$ however, an approximation was needed, as even though the parameter models present in [22] had composition dependence, the interval in which they were valid was too narrow to include these; therefore, a linear approximation of the parameter values was considered with GaAs and $Ga_{0.42}In_{0.58}As$ at extremities.

As for $Al_{0.18}Ga_{0.33}In_{0.49}P$, its contribution is not factored in the results of the present section, since the necessary details could not be found.

**Figure 10:**
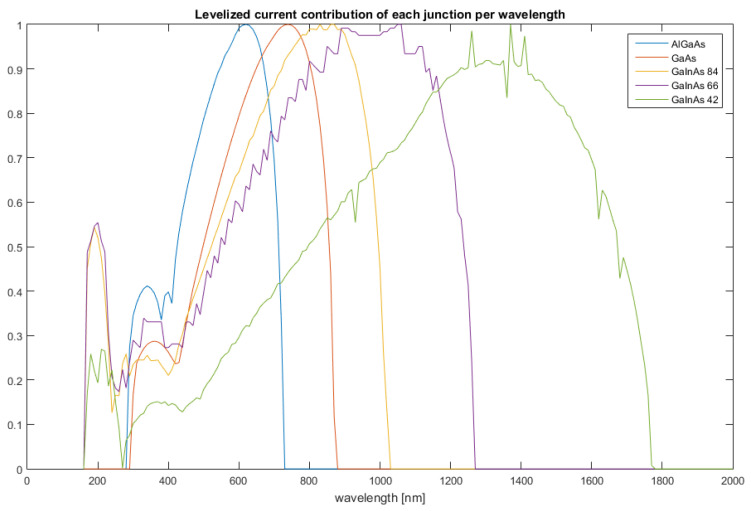
Layer current contribution as a function of wavelength.

Figure 10 displays the results obtained with regard to the levelised contribution to the overall current formation, respective of frequency. In other words, it displays which layer has the prevalent contribution for a given frequency. A closer inspection reveals that each absorber layer displays a peak at a specific frequency; furthermore, the sequence with which the peaks occur corroborates the intended purpose of the layer sequence.

### 3.5. Nanoantenna Model and Parameters

As alluded, a plethora of nanoantenna designs are possible, each with its advantages and disadvantages. In the present work, an aperture nanoantenna was chosen to couple with the multi-junction cell, as it represents one of the simpler designs—and therefore, one of the most reliably reproduced—an important characteristic considering the already extremely complex optical device in which it will be integrated. The nanoantenna design also offers the ability to modify the frequency range with which it interacts via a variation in aperture size and periodicity [23].

**Figure 11:**
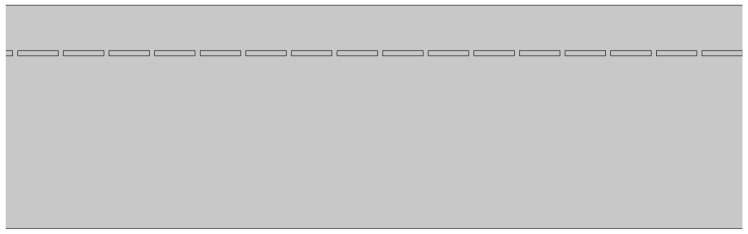

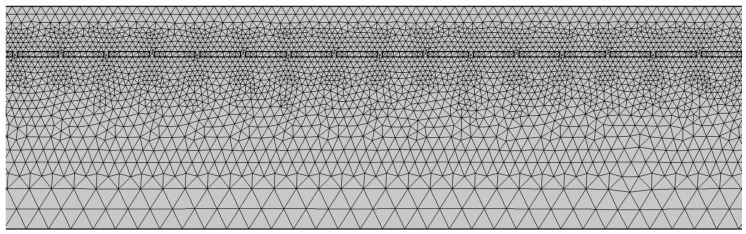
Nanoantenna model and its mesh representation.

Figure 11 displays the nanoantenna array placed in the absorber layer. The array was placed in the first third of the absorber layer with an aperture of 50 nm, spacing of 400 nm and thickness of 50 nm.

### 3.6. Metal Optical Characteristics

**Figure 12:**
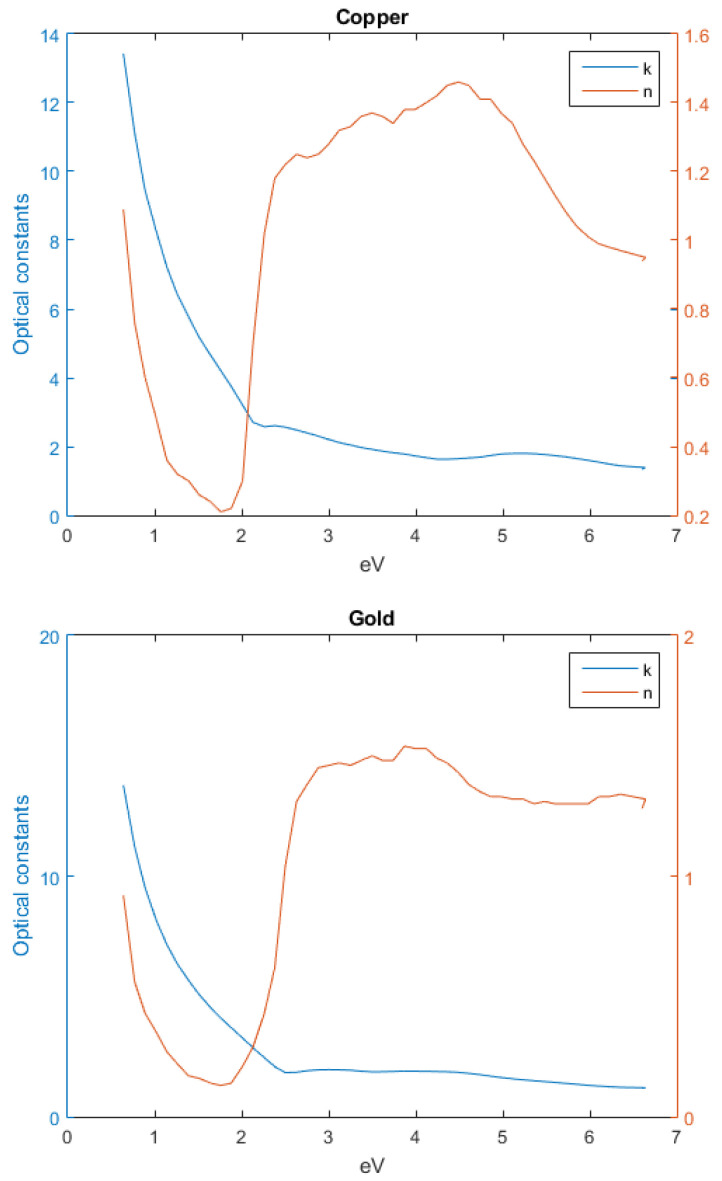

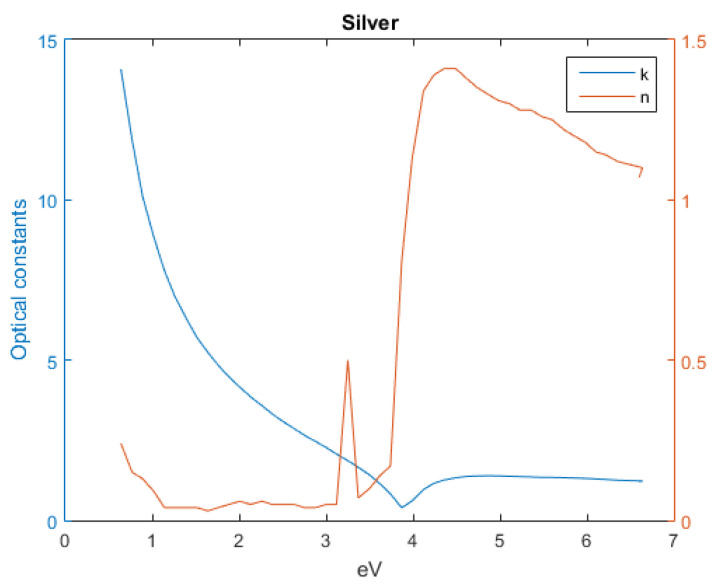
Metals’ optical functions.

An initial assessment of the optical characteristics of gold, silver and copper were performed, in Figure 12, highlighting the influence of n and k parameters, with respect to frequency [24].

From the previous figures, it becomes clear that as the frequency increases, the refractive constant of the metals, n, increases, while the extinction coefficient, k, decreases, corroborating the premise that for higher frequencies, the perfect conductor approximation no longer holds valid, thus displaying a behaviour closer to a dielectric medium.

### 3.7. Photovoltaic Cell with Nanoantenna Array Integration

The final step of the study developed in the present work is the introduction of the nanoantennas in the multi-junction cell, namely, in the absorber layer.

Although the nanoantenna structure is already defined, its composition still remains to be determined at this stage. In order to do so, an optical study should be performed with the full structure.

**Figure 13:**
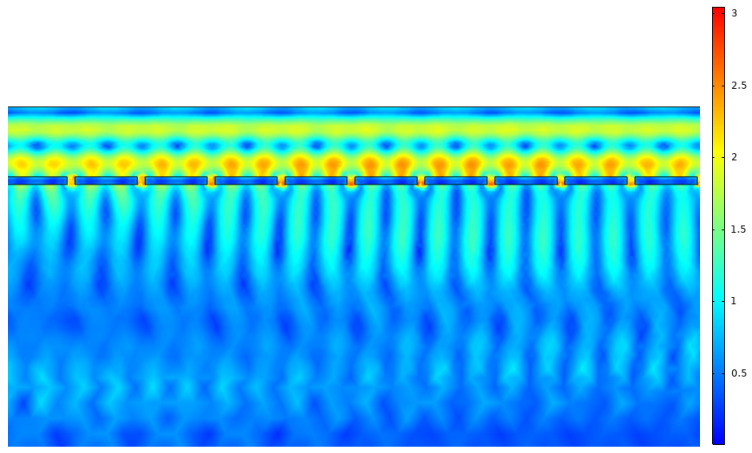
Nanoantenna array influence on the electromagnetic field in the absorber layer.

**Figure 14:**
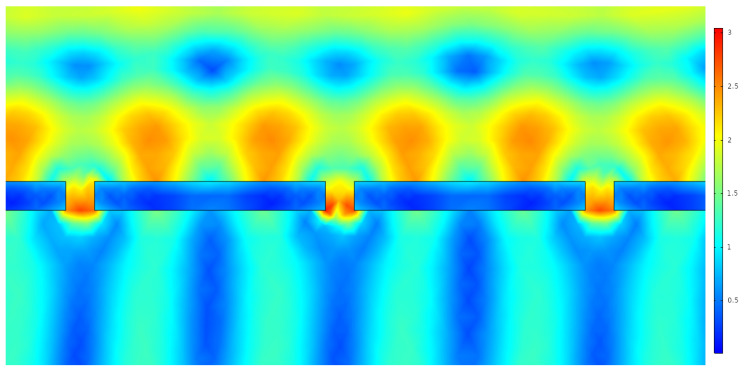
Local field amplification in the nanoantenna apertures.

Figure 13 depicts the GaAs absorber layer, with a copper nanoantenna array. Given that the cell was irradiated with an incident electrical field of unitary norm, the values displayed in the heat map indicate the presence of higher intensity fields. These can be explained with surface plasmons, and with them the extraordinary optical transmission effect. As the cell is irradiated, the photons interact with the valence electrons of the metal, creating plasmons; these plasma oscillations propagate in the surface of the metal and, when coupled with the photons, generate surface plasmon polaritons. These, in turn, due to the shape of the nanoantenna are spacially confined to a specific region, where the field intensity is greater than the incident one, due to these oscillatory phenomena. This spacial field confinement, in aperture nanoantennas, occurs primarily within the aperture, as is made evident in Figure 14.

**Figure 15:**
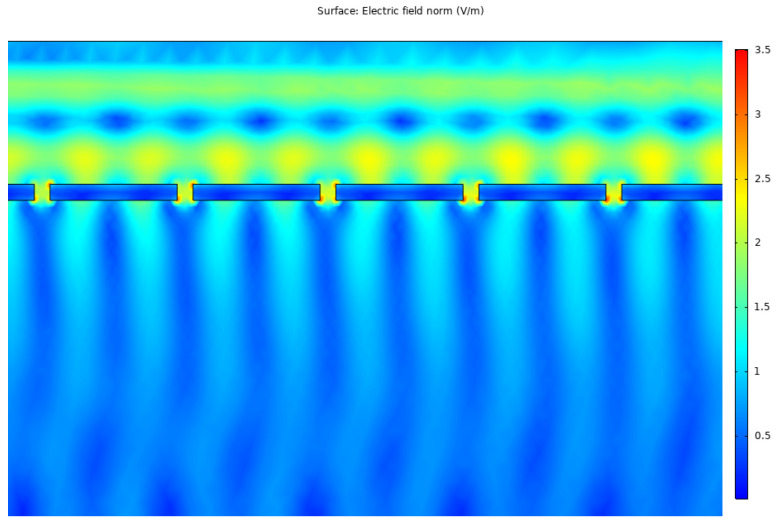
Local field amplification in the nanoantenna apertures for gold array.

**Figure 16:**
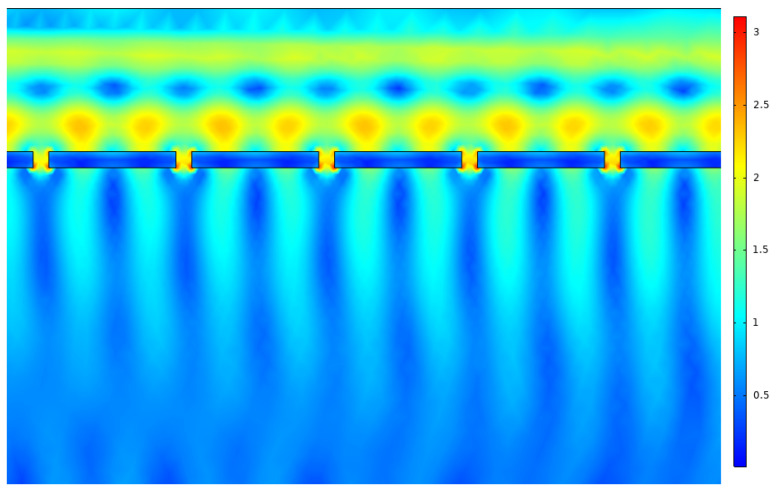
Local field amplification in the nanoantenna apertures for silver array.

Following the results obtained for the copper nanoantenna, a comparison was made with the remaining metals by applying the same conditions, in terms of depth, periodicity and aperture size, for the gold and silver nanoantenna arrays. The results are depicted in Figure 15 and Figure 16.

Although the measured field intensity was higher, it was only so punctually in the case of the gold nanoantenna array, with a decreased performance for the remaining spectral range. Therefore, the metal chosen for the next step of the study was copper. It should be noted that given the required parameters, an approximation was taken, in which the data regarding to carrier mobility and carrier concentration were adapted from an analysis performed on copper thin films [25].

With the nanoantenna array defined, a depth and periodicity sweep was performed in order to correlate these variables with current formation, as well as to display other trends. However, in order to have a basis for comparison, the current generated in the absorber layer as a function of frequency is required.

**Figure 17:**
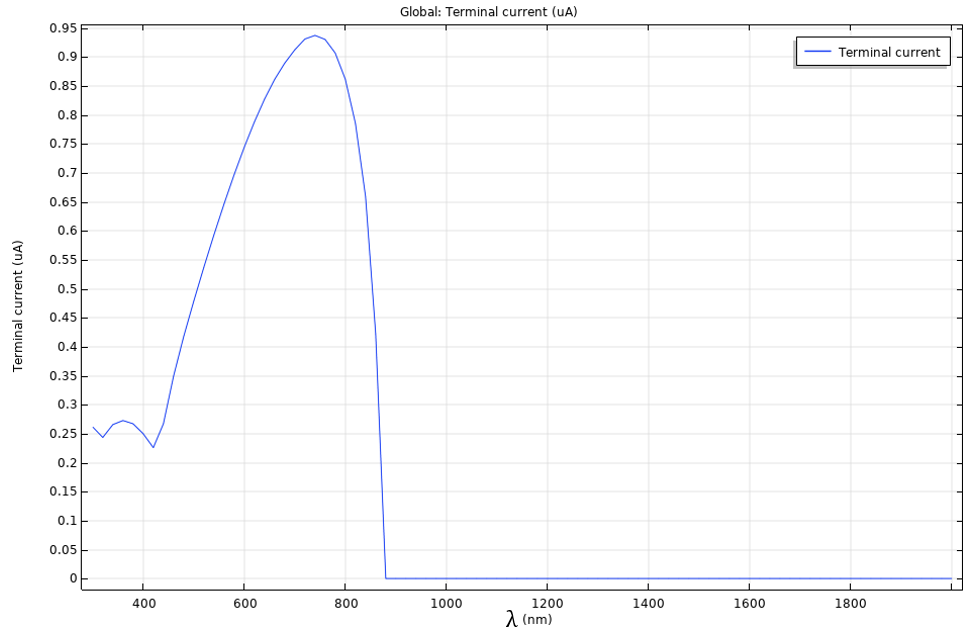
Terminal current measured with respect to frequency for the GaAs absorber layer model, without the nanoantenna.

Figure 17 displays the measured terminal current across the frequency spectrum, from λ = 300 nm to λ = 2000 nm, or from ultraviolet to far into infrared. The current measured at the terminals displays a peak at approximately λ = 740 nm, and an initial no-response zone, the latter being due to the bandgap energy of the semiconductor.

Starting with the depth sweep, the array was placed 1 μm from the surface edge of the absorber layer, and pushed deeper in the layer at increments of 2 μm until it reached 1 μm from the bottom edge. To evaluate the impact that the nanoantenna array had on the cell, the terminal current was measured for each iteration; the results obtained, displayed in Table 1, show the current gain with respect to the reference of Figure 17.

The ratio displayed in Table 1 was obtained through an integration of the measured terminal current across the entire frequency spectrum in study. Comparing the baseline current profile of the absorber layer without the nanoantenna array in Figure 17 to these evidences a clear improvement in overall current formation. This increase in current, could be the result of spacially confining the field in the absorber layer, as it favours the interaction of the incident photons with the charge carriers, especially considering the local increased field values.

Following the depth sweep, a periodicity sweep was performed, in which the aperture size was increased from a base value of 50 nm to 130 nm, with a 5 nm iteration. The baseline depth to place the array was determined with an initial shorter sweep combining the three best results from the previous study with a small aperture variation. Interestingly, the optimum position was further into the absorber layer than Table 1 would suggest, with the depth of 5 μm ultimately being the choice for the extensive periodicity sweep.

Table 2 displays the ratio of the terminal current integral, for a nanoantenna array placed near the surface edge of the layer. Although promising results, as the ratios clearly show an improvement in the overall current formation across the entire frequency spectrum, they are inferior to the results obtained when the array was placed deeper, and were only included to evidence the depth baseline choice.

From the ratios displayed either in Table 2 or Table 3, a significant improvement in the overall current formation is evident, although with greater results when the array was placed deeper into the absorber layer, as already stated. The peak was observed with a gap of 130 nm; however, the variation did not display a linear relation with the aperture size, as similar results were obtained from 95 nm to 105 nm.

As previously mentioned, additional trends displayed as the nanoantenna array was swept in terms of depth and periodicity were of interest as well. In that regard, it was evidenced that not only did the current increase with the introduction of the nanoantenna array, the position of the peak could also suffer changes.

**Figure 18:**
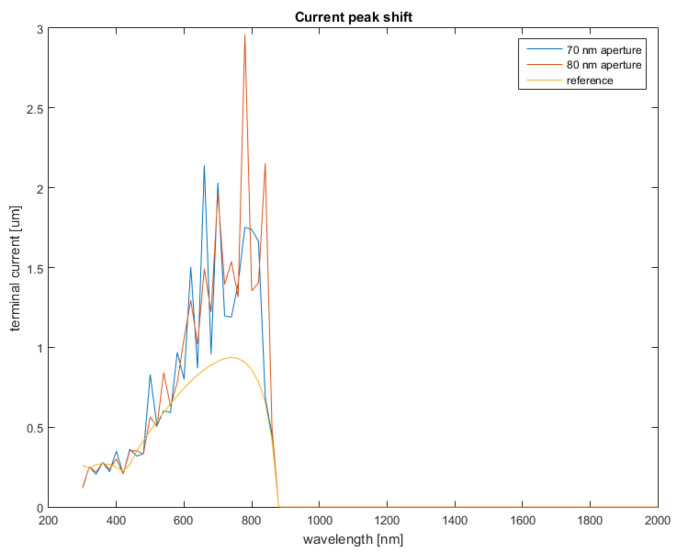
Current obtained on the layer’s terminal for each considered aperture size.

Figure 18 displays the reference current profile, on which two additional profiles were overlaid, corresponding to two different aperture sizes. In the case of the 70 nm aperture gap, the peak current is obtained when the wavelength is approximately λ = 660 nm, and in the case of 80 nm, the peak current is obtained at a wavelength of approximately λ = 790 nm. Given the reference peak at λ = 740 nm, both results represent a significant shift in frequency, corresponding to a blue shift and a red shift, respectively.

Frequency shifts were not unexpected, as previous works already hinted at the structural parameters of aperture nanoantennas having an effect on the output frequency [23]. However, this opens up the possibility for a series of interesting applications when applied to PV cells, as a frequency shift could provide a means to alter the absorption profile of the cell.

## 4. Conclusions and Closing Remarks

An initial theoretical basis was presented, with the intent of displaying the trends and evolution of the PV technologies available today, where it was shown that even though multi-junction devices are the champions in terms of power conversion efficiency, their use is still limited by its complex and costly fabrication process. Another optical device was then introduced, the nanoantennas, which show great potential for future applications, although currently constrained by the achievable rectifying speed. These aspects were at the origin of this work, as the two technologies displayed great potential with several characteristics that perhaps combined could lead to an interesting result.

The present work had, as its core objective, a behaviour study into multi-junction PV cells and nanoantennas, tying these two with the intent of studying their interaction. This was then divided into several stages, each with a separate objective, comprising the several sections of this work, from the optical study of each device to a more extensive optoelectronic analysis that combined the two.

With respect to the behaviour analysis of the multi-junction PV device, a viable optoelectronic basis model was developed. This is particularly visible in the results shown in Figure 10, where the current contributions of each layer are clearly defined with consecutive peaks, a definite characteristic of the device. Another important aspect was the field interaction with the layers, as each displayed a characteristic absorption profile, with clear frequency boundaries, which is tied with their intended placement in the device.

The optical analysis of the nanoantenna array had first as its objective defining the metal that would constitute the array to later couple with the cell. From the results obtained, and given the layer chosen, copper showed the best results. Along with this, it was possible to demonstrate the extraordinary optical transmission phenomenon, validated through increased output field intensity measurements, with a clear peak value in the aperture. These oscillatory phenomena, from plasmon and photon coupling, are clearly displayed in Figure 13 and Figure 14.

When coupled with the nanoantenna array, the PV cell showed great improvements in terms of current formation. However, from the sweeps performed and the results obtained, no clear correlation with aperture size could be defined. Nonetheless, as demonstrated by the results displayed in Table 2 and Table 3, even if the structural parameters of the array are not optimised, the improvement in current still is clear and relevant.

Without dismissing improvements in the order of the ones shown in Table 1, Table 2 and Table 3, which require further attention as to what it could lead to in regard to PV cells, the most exciting phenomena shown is the prospect of shifting the absorption profile of a layer. This is particularly interesting as it would allow for tuning the absorption spectrum of a cell to a given spectral range. Further, when applied specifically to multi-junction devices, this ability could allow for swapping out complex semiconductor compounds with other cheaper and more readily available alternatives as well as diminishing the overall cost of the cell with said swap. This price decrease would in turn increase the viability of technology and open it to uses beyond space applications.

## Figures and Tables

**Table 1:** Depth sweep with terminal current ratio.

Depth	$\frac{I}{I_{\mathrm{ref}}}$
**Ref**	**1**
1	2.3284
3	2.1323
5	2.0176
7	1.7460
9	1.5409
11	1.2213
13	1.0695
15	0.8624

**Table 2:** Periodicity sweep at 1 μm with terminal current ratio.

Aperture Size	$\frac{I}{I_{\mathrm{ref}}}$
**Ref**	**1**
70 nm	1.0538
75 nm	1.1228
80 nm	1.2025
85 nm	1.1427
90 nm	1.2996
95 nm	1.4011
100 nm	1.3213

**Table 3:** Periodicity sweep at 5 μm with terminal current ratio.

Aperture Size	$\frac{I}{I_{\mathrm{ref}}}$
**Ref**	**1**
50 nm	1.2213
55 nm	1.2579
60 nm	1.3204
65 nm	1.3994
70 nm	1.4319
75 nm	1.4525
80 nm	1.5545
85 nm	1.4146
90 nm	1.5862
95 nm	1.6294
100 nm	1.6244
105 nm	1.6485
110 nm	1.5838
115 nm	1.5342
120 nm	1.5537
125 nm	1.4525
130 nm	1.6976

**Figure A1:**
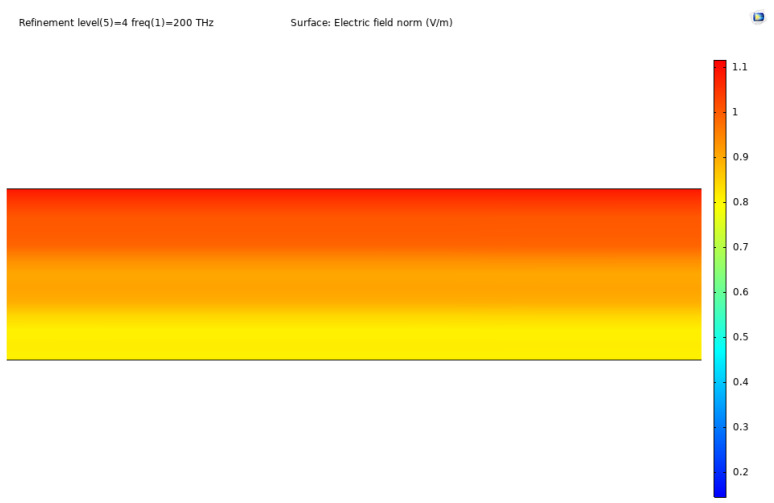

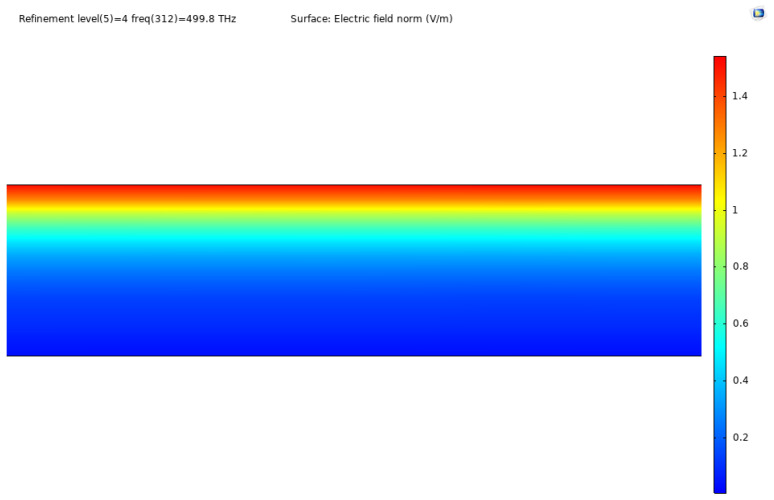
Electric field norm of $Al_{0.18}Ga_{0.33}In_{0.49}P$ at 200 THz and 500 THz.

**Figure A2:**
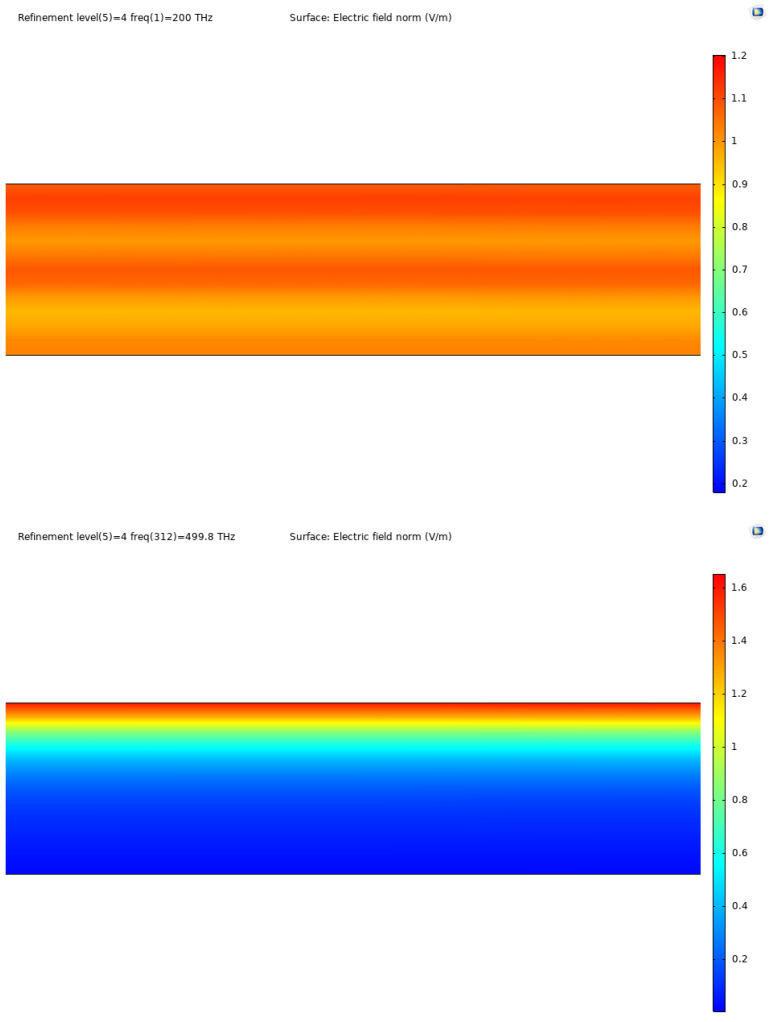
Electric field norm of GaAs at 200 THz and 500 THz.

**Figure A3:**
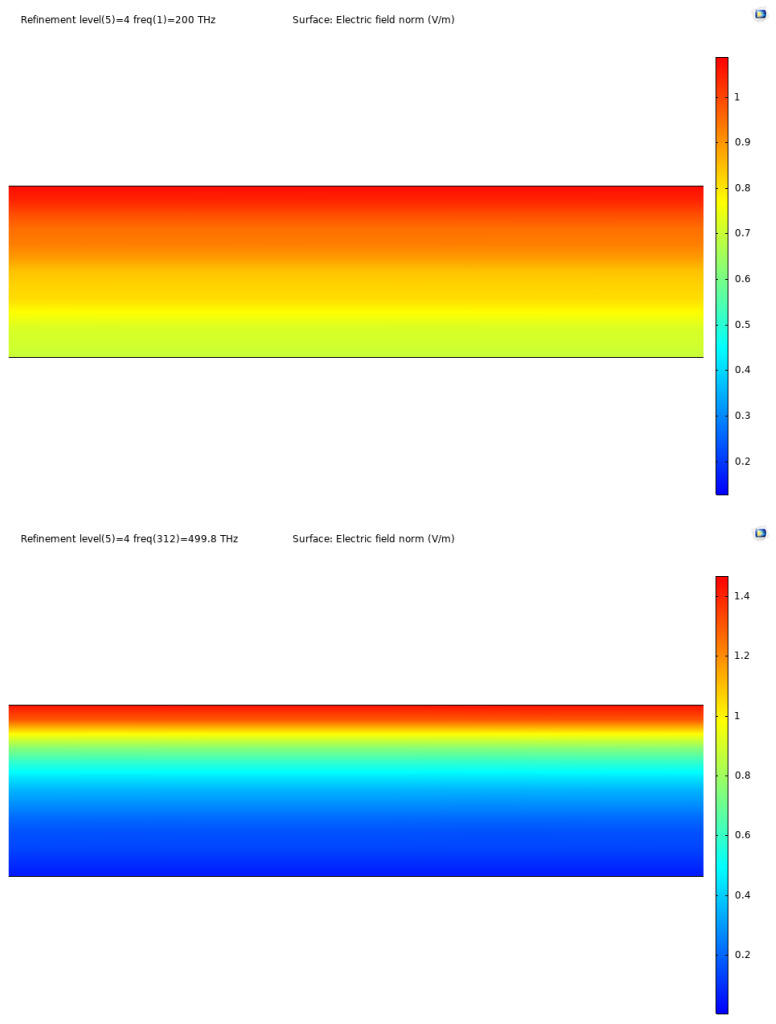
Electric field norm of $Ga_{0.84}In_{0.16}As$ at 200 THz and 500 THz.

## Data Availability

Not applicable.

## Appendix A

Figure A1, Figure A2, Figure A3, Figure A4 and Figure A5 show the obtained radiation profile for the other semiconductors’ layers.
